# Comparative analysis of nucleotide translocation through protein nanopores using steered molecular dynamics and an adaptive biasing force

**DOI:** 10.1002/jcc.23525

**Published:** 2014-01-09

**Authors:** Hugh S C Martin, Shantenu Jha, Peter V Coveney

**Affiliations:** [a] Department of Chemistry, Centre for Computational ScienceUCL, 20 Gordon Street, London, United Kingdom; [b] Department of Electrical Engineering BuildingRutgers, 94 Brett Road, Piscataway, New Jersey

**Keywords:** nanopore, molecular dynamics, adaptive biasing force, protein, DNA

## Abstract

The translocation of nucleotide molecules across biological and synthetic nanopores has attracted attention as a next generation technique for sequencing DNA. Computer simulations have the ability to provide atomistic-level insight into important states and processes, delivering a means to develop a fundamental understanding of the translocation event, for example, by extracting the free energy of the process. Even with current supercomputing facilities, the simulation of many-atom systems in fine detail is limited to shorter timescales than the real events they attempt to recreate. This imposes the need for enhanced simulation techniques that expand the scope of investigation in a given timeframe. There are numerous free energy calculation and translocation methodologies available, and it is by no means clear which method is best applied to a particular problem. This article explores the use of two popular free energy calculation methodologies in a nucleotide-nanopore translocation system, using the α-hemolysin nanopore. The first uses constant velocity-steered molecular dynamics (cv-SMD) in conjunction with Jarzynski's equality. The second applies an adaptive biasing force (ABF), which has not previously been applied to the nucleotide-nanpore system. The purpose of this study is to provide a comprehensive comparison of these methodologies, allowing for a detailed comparative assessment of the scientific merits, the computational cost, and the statistical quality of the data obtained from each technique. We find that the ABF method produces results that are closer to experimental measurements than those from cv-SMD, whereas the net errors are smaller for the same computational cost. © 2014 The Authors Journal of Computational Chemistry Published by Wiley Periodicals, Inc.

## Introduction

The translocation of nucleic acid strands through confined protein pores has substantial biological relevance, for example, the transfer of antibiotic resistance genes between bacteria,^1^–^3^ phageinfection,^4^ and the uptake of oligonucleotides into kidney tissue.^5^ Moreover, the passage of nucleic acids through pores is also of biotechnological and diagnostic relevance; for these applications, a single nanopore is inserted into a lipid bilayer, and individual negatively charged nucleic acids are electrophoretically driven through the pore. The passage of strands leads to detectable fluctuations in the ionic pore current. Data from these single channel current recording (SCCR) experiments provide information on polymer length, orientation, and composition for polymers such as single-stranded DNA and RNA.^6^–^11^ The capability of SCCR to reveal information on translocating DNA strands has long been under investigation as an avenue for faster and cheaper genetic sequencing.^12^ In recent years, it has been demonstrated that SCCR has sequencing capabilities,^13^,^14^ and in February 2012, Oxford Nanopore Technologies demonstrated a fully functional genetic sequencing device, expected to be available commercially soon.^15^

Understanding the microscopic processes of nucleic acid translocation through nanopores is crucial in improving SCCR techniques and apparatus for sequencing DNA. Using molecular dynamics (MD) simulations of the translocation process, it is possible to retrieve kinetic and structural information that cannot be obtained solely through experiment. Experiments investigating the translocation of nucleic acid under the influence of a transmembrane potential indicate that the process typically takes hundred of microseconds to tens of milliseconds.^7^ However, accurately simulating biological processes and systems with atomistic resolution remains a challenge for many reasons, not least of which are the substantial computational resources required. Even with state-of-the-art high-end computers, performing simulations with atomistic resolution for such large systems over the required time-scales remains infeasible at present. Simple approaches to circumventing this issue can give rise to undesirable consequences––for example, the application of an artificially high transmembrane potential to induce faster translocation causes disruption of the lipid membrane; applying a high uniform electrostatic field to only the translocating atoms fails to translocate nucleic acid polymers through the protein nanopore.^16^ Thus, if these events are to be effectively investigated using simulation, novel approaches and better algorithms are required in order to bridge the gap between time-scales over which the translocation events occur and those that are accessible using simple equilibrium simulations.

Free energy changes associated with chemical processes frequently provide important insights. By computing the free energy difference associated with a change of state, it is often possible to establish stable states, their thermodynamics properties, the kinetics of transitions between states, and indeed to infer how stable states are altered by external conditions. Such changes of state include protein mutation, protein-ligand binding, conformational changes, and molecule translocation. It is, of course, both possible and valuable to calculate experimental free energy changes, and there has recently been a considerable amount of research dedicated to comparing experimental and theoretical free energy changes.

There are several well established methods for extracting free energy from MD simulations. These include history-dependent methods such as metadynamics,^17^ self-healing umbrella sampling,^18^ and the adaptive biasing force method (ABF),^19^ which can bias a translocating molecule along a reaction coordinate. Other methods such as constant velocity-steered MD (cv-SMD) or constant force-steered MD^20^ may be used to entice a molecule along a reaction coordinate, based on the behavior of which free energy calculation methods such as Jarzynski's equality (JE)^21^ or Crooks fluctuation theorem^22^ may be used to extract the free energy.

Cv-SMD/JE and the ABF methodology are two well-established and widely used translocation/free energy calculation methods that serve as exemplary methodologies for the purposes of such a comparison. The methodologies have key similarities, yet important differences in their “dynamics.” It is the aim of this article to explore these similarities and differences. We believe that conclusions from this investigation can be extrapolated to many other translocation and free energy calculation methods.

In cv-SMD, the translocating molecule of interest is attached via a harmonic spring to a point in space that is pulled at constant velocity. Using the force experienced by the spring, the free energy of translocation may be determined using JE to equate the free energy to the work done. In the ABF methodology, the translocating molecule of interest is encouraged along the reaction coordinate by introducing a biasing force into the equations of motion for an atom or group of atoms in the molecule. This biasing force opposes the free energy estimate for a section of the reaction coordinate and is calculated using the instantaneous forces acting on the atom(s) in question. See Supporting Information for a more detailed account of the theoretical background to these two methodologies.

A major benefit of algorithms such as cv-SMD and ABF is that they permit larger and/or more complex systems to be investigated using a given computational budget (comprising the hardware and computational hours available). It is, therefore, pertinent to choose a system of considerable size and complexity for this study, as the behavior of the algorithms at these limits has been hitherto unclear. The system should also have experimental or biological relevance, in order that we may draw comparisons with experimental data, and any insight we gain may have relevance to other studies and future research.

**Figure 1:**
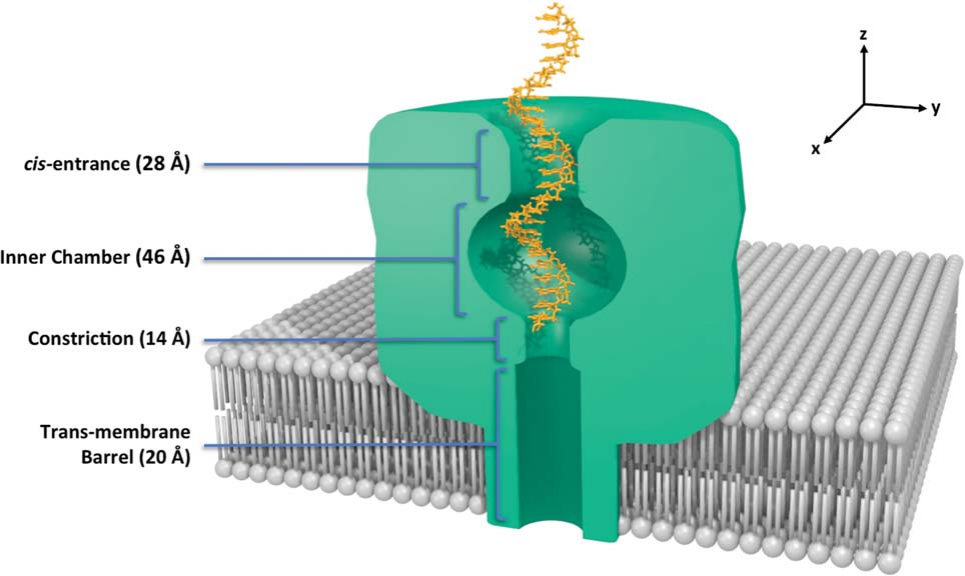
Figure representing a cross-section of the protein pore αHL, and the starting configuration for the simulations studied here. The heptameric αHL protein pore (green) is inserted into a lipid bilayer (gray). The *cis*-entrance at the top of the protein pore is about 28 Å in diameter and the *trans-*entrance at the bottom of the pore is about 20 Å. Key features inside the pore interior include the wide inner chamber (up to 46 Å wide), a constriction about half way down the pore (14 Å wide), followed by the transmembrane barrel (20 Å wide) that spans the lipid bilayer. The translocating molecule, in this example a polynucleotide (orange), is positioned with the 3′-end at the top of the constriction.

The translocation system we have chosen to investigate is the passage of nucleotide molecules through the protein nanopore α-hemolysin (αHL), depicted in Figure 1. αHL is a heptameric protein-pore that has been extensively studied in experiments and computer simulations,^10^,^11^,^16^,^23^–^30^ and is the biological pore currently in use in the developing technologies at Oxford Nanopore.^15^ We explore the protein-pore translocation of the nucleic acid strands polyadenosine, poly(A), and polydeoxycytidine, poly(dC), which are single strands of RNA and DNA, respectively. Poly(A) and poly(dC) molecules of 100–200 bases in length exhibit a 20-fold difference in translocation time through αHL in SCCR experiments.^7^ We also translocate single nucleotides A_1_ and dC_1_ to discern their relative contributions to the free energy profiles.

The shape of the pore shown in Figure 1 indicates the steric barriers that a translocating polynucleotide will encounter, the most significant of these being the constriction half way through the pore. Here, secondary structure conformations such as helical conformations will need to unwind for translocation to be permitted. In addition to steric factors, there are also electrostatic interactions to consider. We recently published an investigation into the nucleotide-nanopore system using cv-SMD/JE.^31^ The study applied the cv-SMD translocation technique in a system of unprecedented size, revealing new insight into the translocation process. In that study, we identified the existence and significance of a phosphate-lysine interaction. Bond et al. have since verified this interaction in a separate study.^32^ They performed nucleotide translocation simulations through a simplified αHL pore using an applied transmembrane potential and determined that the phosphate-lysine interaction plays a major role. In fact there are 11 positively charged residues at the surface of the protein (and are accessible to a translocating molecule) that may pose a barrier to translocation; these are lysine residues 8, 21, 46, 51, 131, 147, 154, 237, 288, and arginine residues 56 and 104. It is Lys-147 at the constriction that is the most significant, its impact being enhanced by the tight diameter of the pore where it resides.

In this article, we use the ABF methodology to investigate the nucleotide-nanopore system and provide a comprehensive comparison of the two methodologies. By performing simulations using cv-SMD/ JE and ABF under comparable conditions, we are able to make direct comparisons of the data quality and associated errors, the modes of translocation, the free energy calculations, and the computational resources that each method requires.

It should be noted that there are a developing wealth of options for computational scientists wishing to explore nucleotide translocation through αHL. Recent advances in simulated translocation techniques such as Grid-SMD have opened the door to speed up steady-state translocation, which permits conditions very close to those found in experiment.^16^ This, combined with modern supercomputing infrastructure (such as Anton^33^) boasting substantially enhanced computing power, means that it is now in principle feasible to attack the nucleotide/αHL problem with brute force, running a full translocation event under desirable conditions which do not involve the same assumptions and approximations that cv-SMD and ABF impose, though this has yet to be done with αHL.^34^ Due to the considerable computational cost of such simulations, and the continuing need for larger scale simulations, nonequilibrium translocation techniques such as ABF and cv-SMD will remain in common use, and our article is concerned with the application of these techniques to larger scales and to compare them. As discussed in our previous work,^31^ we have focused our investigations on a partial translocation through a section of the pore interior representing the dominant barrier to translocation. Although this does not allow direct comparison with experiment, it allows us to explore the key part of the pore while producing statistically meaningful results with which to compare the two translocation methodologies.

In the Section “Method”, we provide details of the model and techniques used to perform our simulations. In the Sections “Adenine and Deoxycytosine Translocation Using cv-SMD and Adenine” and “Adenine and Deoxycytosine Translocation Using an ABF”, we present analyses of simulations of single and polynucleotide translocation through wild type αHL, for cv-SMD, and ABF, respectively. In the Section “Comparison of cv-SMD with the ABF Method”, we compare cv-SMD/JE to ABF for the nucleotide-nanopore system. In the final section, we present our conclusions.

## Method

Martin et al. describe the details of the model construction and simulation parameters.^31^ The cv-SMD method section described there^31^ is applicable to the cv-SMD simulations in this article; therefore, only an overview of this method, along with some additional points of note, will be provided here. For the ABF simulations reported in this article, the majority of the parameters and model construction from the cv-SMD method also apply, with some key exceptions. In this section, we describe and justify the ABF-specific parameters that we have chosen and explored.

The αHL crystallographic structure coordinates were taken from Protein Data Bank (PDB) entry 7AHL. The protein was inserted into a patch of 150 Å × 150 Å pre-equilibrated and solvated 1-palmitoyl-2-oleoyl-sn-glycero-3-phosphocholine lipid bilayer using the Visual Molecular Dynamics (VMD) plug-in membrane, aligned to the *xy*-plane. The center of mass of the hydrophobic belt of αHL (residues 118–126 and 132–142) was aligned with the center of mass of the lipid bilayer. The system was solvated in a water box of pre-equilibrated water molecules and the aqueous solution was set at 1M NaCl. Figure 1 shows αHL inserted in a lipid membrane as it appears in our models. The protonation states chosen are consistent with the typical SCCR recording pH range of around pH 8.0.^6^,^11^ Key protonation states include: protonated, positively charged amine groups of lysine and arginine residues; unprotonated, negatively charged interchain phosphate groups; and unprotonated, doubly negatively charged terminal phosphate groups on the single nucleotide molecules.

The poly(A) and poly(dC) molecules were constructed using the AMBER module *nucgen*^35^ to 25 bases in length. Single nucleotide PDB files of adenosine (A_1_) and deoxycytidine (dC_1_) monophosphates were obtained from the PDB (PDB identifiers AMP and DCM, respectively). The topology files were modified accordingly to produce accompanying Protein Structure File (PSF) files. The final models consisted of 328,000 and 262,000 atoms for the 25-base polynucleotide and single nucleotide models, respectively. The nucleotide molecules were orientated with the C3′-carbon atom of the leading residue was aligned with the center of the alpha carbon atoms (C_α_) of protein residue 111. The nucleotide molecules were pulled or biased from this starting position toward the *trans*-entrance of the pore. A partial translocation of the leading residue through the constriction was performed to maximize the number of translocation samples performed given a finite computational budget; Martin et al. justify this selection of reaction coordinate in detail.^31^ An example of the starting position of the polynucleotides is shown in Figure 1.

Simulations were performed using the MD simulation package NAMD version 2.71b.^36^ The CHARMM^37^ force field was applied using all-hydrogen parameter files for CHARMM22 proteins and CHARMM27 lipids and nucleic acids.

To gather a set of samples to form an ensemble, multiple simulations of the nucleic acid molecule translocating past the same section of the pore were required. The initial configurations used to perform these translocation samples were obtained by capturing snapshots of the atomic positions and velocities, separated by 0.2 ns at equilibrium, with the SMD atom position fixed and the C_α_ protein atoms restrained.

Unless otherwise stated, harmonic constraints of 0.5 N/m were placed on the C_α_ atoms of the protein amino acid residues to prevent translocation of the protein. In cv-SMD simulations, this allows the reaction coordinate to indicate specific protein-nucleic acid interactions. In ABF simulations of the type described in this article, the relationship between the reaction coordinate and the pore interior is maintained regardless of shifts in the protein's location.

### cv-SMD method

For cv-SMD translocation, the full reaction coordinate was explored using several 1-ns simulations in sequence. An overlap of 0.2 ns between sequential simulations was performed to enable the removal of start-up artifacts. The SMD atom was pulled at 0.04 Å/ps and the SMD spring constant was set to 100 kcal/mol, which Martin et al.^31^ established as suitable for these molecular models.

### ABF method

The biasing force was applied to the C3′-atom of the leading residue. The ABF implemented using the *colvar* module.^38^ Force measurements were accumulated in bins of 0.25 Å (unless otherwise stated) for 16-Å length trajectories. The reaction coordinate in the ABF methodology was calculated as a function of distance from the translocating molecule to a reference set of atoms in the protein-pore (Glu-111), in contrast to cv-SMD. This relative definition of the reaction coordinate allows for the protein to be left unconstrained in ABF simulations. Unless otherwise stated, the simulations reported in this section were performed with an unconstrained αHL pore.

Although only the *z*-axis separation was controlled by the biasing force, the steric constraints of the pore interior were sufficient to keep each sample trajectory within the desired *xy*-boundaries. The biased atom was kept within the outer *z*-axis boundaries of the reaction coordinate by a harmonic force implemented at either end.

The length of the reaction coordinate was set to 16 Å, spanning the length of the constriction. It is possible to split reaction coordinates into segments and construct free energy profiles from each of the segments. Splitting the reaction coordinate can help prevent the biased atom getting stuck. However, this is not necessary with a reaction coordinate length as short as 16 Å. Furthermore, introducing too many segments can cause the harmonic restraints at the ends of each segment to significantly impact the free energy values, and so it should be implemented with caution. The number of simulated timesteps required to sample the reaction coordinate depends on the force measurement threshold parameter and the diffusion time, which varies between simulations. The simulations were, therefore, performed in blocks of 100,000 to 1 million MD integration timesteps until the full reaction coordinate was sampled. The force measurements threshold parameter (*ζ*) are investigated in Supporting Information.

### Summary of the models and simulations performed

This subsection summarizes the key configuration details in the simulations represented in this article. This is presented in the form of a table (Table1), in order for the reader to be able to quickly refer to and understand our data, particularly when comparisons are being drawn between multiple figures.

**Table 1:** Table listing key components of the simulated systems from the profiles in this article.

Configuration name	Pulling method	Nucleotide base	Nucleotide bases	Samples performed	Protein constraints	ABF threshold	Translocation distance (Å)	Figure number
A_25_-cvSMD-48Å	cv-SMD	Adenine	25	16	Constrained	N/A	48	2
dC_25_-cvSMD-48Å	cv-SMD	Deoxycytosine	25	16	Constrained	N/A	48	
A_1_-cvSMD	cv-SMD	Adenine	1	16	Constrained	N/A	16	3
dC_1_-cvSMD	cv-SMD	Deoxycytosine	1	16	Constrained	N/A	16	
A_25_-ABF-20k	ABF	Adenine	25	4	Unconstrained	20,000	16	4
dC_25_-ABF-20k	ABF	Deoxycytosine	25	4	Unconstrained	20,000	16	
A_1_-ABF-5k	ABF	Adenine	1	4	Unconstrained	5000	16	5
dC_1_-ABF-5k	ABF	Deoxycytosine	1	4	Unconstrained	5000	16	
A_25_-cvSMD-16Å-4s	cv-SMD	Adenine	25	4	Constrained	N/A	16	6a
dC_25_-cvSMD-16Å-4s	cv-SMD	Deoxycytosine	25	4	Constrained	N/A	16	
A_25_-ABF-16Å-4s	ABF	Adenine	25	4	Constrained	5000	16	6b
dC_25_-ABF-16Å-4s	ABF	Deoxycytosine	25	4	Constrained	5000	16	

## Adenine and Deoxycytosine Translocation Using cv-SMD

In our previous study,^31^ we used single nucleotides and polynucleotides in wild type and mutated αHL nanopores to gain insight into the translocation process. We found that a phosphate-lysine electrostatic interaction at the pore constriction played a key role in translocation, proving its significance by mutating the lysine residue in question, which significantly impacted the free energy profiles. The extent to which this interaction occurred for a particular nucleotide molecule was highlighted as a potential cause for the discrimination of poly(A) and poly(dC) translocation. With a demonstrated dependence of the interaction on local solvation ionic environments, it was deemed necessary to increase the sampling of the reaction coordinate to give dependable insight.

In this section, we extend our previous investigation by comparing the free energy profiles with significantly greater sampling for A_25_, dC_25_, A_1_, and dC_1_ translocation through wild type αHL using cv-SMD. By providing a set of highly sampled profiles in this way, we can use the dataset as a reference point for the validation of new data that has not been permitted the same sampling budget.

### Polynucleotides in wild type αHL

**Figure 2:**
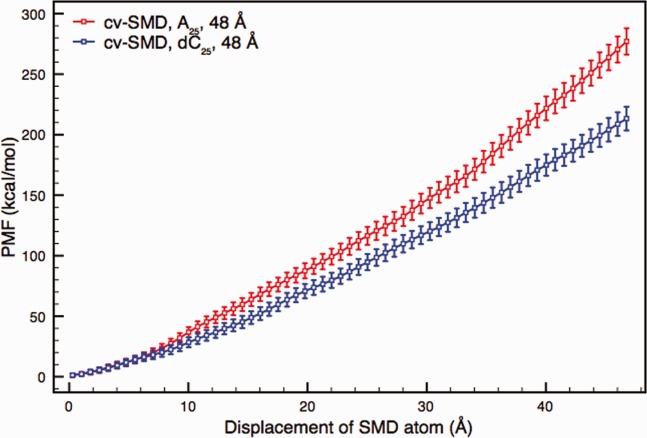
Free energy profiles of A_25_ and dC_25_ translocation from a set of cv-SMD simulations. The reaction coordinate spans 48 Å from the top of the constriction to the bottom of the *trans*-entrance of wild type αHL. Each profile was derived from 16 samples, calculated using a bin width of 0.75 Å. The free energy estimate for A_25_ is approximately 30% higher than that of dC_25_ at the end of the 48 Å reaction coordinate. The plots show discrimination of A_25_ and dC_25_ with nonoverlapping error bars after 11 Å of translocation. [Color figure can be viewed in the online issue, which is available at http://wileyonlinelibrary.com.]

The translocation of poly(A) and poly(dC) is shown in Figure 2. The figure shows the free energy profiles for a translocation over 48 Å for A_25_ and dC_25_ with 16 samples used for the calculation of each profile. Here, the SMD atom at the 3′-end of the nucleic acid polymer was pulled from the top of the constriction to the bottom of the transmembrane barrel. Given the pore dimensions, as listed in Figure 1, the steric barriers to translocation occur mainly within this region.

The free energy plots from Figure 2 show that A_25_ displays a higher free energy profile than dC_25_, with nonoverlapping error bars from 11 Å onward. The separation between the profiles continues to grow throughout the translocation process with the free energy estimate for A_25_ being approximately 30% higher than that of dC_25_ at the end of the 48 Å reaction coordinate. The higher free energy values for A_25_ compared to dC_25_ is in qualitative agreement with the longer experimental translocation times for A_25_.^7^

### Single nucleotides in wild type αHL

Using single nucleotide translocation simulations, we can obtain a clear picture as to what kind of molecular interactions give rise to energy barriers to translocation. This is because the contributions to translocation barriers are reduced to those attributable to the small molecule, whose size and relative simplicity make it straight forward to inspect visually. With a polymeric molecule, numerous steric and electrostatic interactions occur along its length, making it difficult to identify major points of interest. By comparing the single nucleotide to polynucleotide translocation, we can also infer the degree to which nonequilibrium effects impact the polynucleotide free energy profiles.

**Figure 3:**
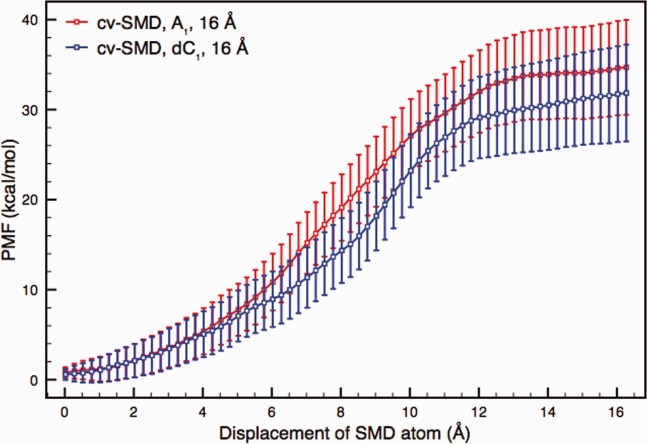
Free energy profiles of A_1_ and dC_1_ translocation from a set of cv-SMD simulations. The reaction coordinate spans 16 Å through the constriction of wild type αHL. Each profile was derived from 16 samples, calculated using a bin width of 0.25 Å. The two free energy profiles do not show discrimination outside of the error bars. Compared to the polynucleotide profiles, the single nucleotide profiles exhibit a distinctive shape, showing a rapid rise of gradient after 4 Å, then a large reduction in gradient after 10 Å, effectively leveling off. This corresponds to steric and electrostatic interactions reaching a maximum between 4 and 10 Å; thereafter the molecule exits the constriction into the wider uncharged transmembrane barrel, giving little resistance to ongoing translocation.

Figure 3 shows the free energy profiles from A_1_ and dC_1_ translocation through wild type αHL. Our previous study^31^ showed that an electrostatic interaction between the nucleotide phosphate (negatively charged) and the protein lysine 147 (positively charged) skewed the values of these single nucleotide profiles in unexpected ways. The result of this is higher free energy values in the dC_1_ profile due a particularly strong phosphate-lysine contribution. The consequence of the upshifted dC_1_ profile is the barely distinguishable A_1_ and dC_1_ profiles shown in Figure 3.

The phosphate-lysine interaction and the small size of the single nucleotide molecule contribute to the distinct profile shape we see in Figure 3. Compared to the polynucleotide profiles, which exhibit a relatively consistent gradient throughout the reaction coordinate, both single nucleotide profiles exhibit a distinctive curve. The single nucleotide profiles show a rapid rise of gradient after 4 Å, then a significant reduction in the gradient after 10 Å, effectively leveling off. This shape corresponds to steric and electrostatic interactions reaching a maximum between 4 and 10 Å of translocation, after which the nucleotide molecule exits the constriction into the wider and uncharged transmembrane barrel, offering little resistance to ongoing translocation.

## Adenine and Deoxycytosine Translocation Using an ABF

In this section, we use ABF as an alternative means of investigating the translocation process. As the ABF method has not been used for this system previously, it is important to fully establish the optimum parameters, and validate the results in comparison to the heavily sampled cv-SMD data, as well as in comparison to experimental findings. We examine A_25_, dC_25_, A_1_, and dC_1_ translocation using ABF and find that it qualitatively reproduces the experimental findings of poly(A) and poly(dC) translocation and the major observations from the highly sampled cv-SMD data.

### Polynucleotide translocation with an ABF

As with the cv-SMD simulations in Figure 3, our primary measure of the validity of these simulations is the reproduction of qualitative experimental findings from poly(A) and poly(dC) translocation through αHL. This subsection presents multisample free energy profiles for the 16 Å reaction coordinates of A_25_ and dC_25_. As we saw in the cv-SMD investigation, 16 Å is sufficient to examine translocation at the pore-constriction, and doing so allows for well sampled data within a reasonable computational budget and time-frame.

In Supporting Information, we explore the interesting option of leaving the protein completely unconstrained, which is permitted due to the nature of the calculation of the reaction coordinate in ABF simulations. The investigation shows that constraining the protein leads to smaller errors at the expense of moving further from experimental conditions. We therefore proceeded with an unconstrained protein when investigating ABF alone, and a constrained protein when comparing ABF to cv-SMD.

Another major parameter of particular interest in ABF simulations is the value of *ζ*. This has a major impact on the translocation time and the influence of nonequilibrium effects; we, therefore, investigate this parameter in great detail in Supporting Information. Our investigations show that simulations where *ζ* = 5000 are expected to contain a significant degree of nonequilibrium contributions in the free energy profiles. At *ζ* = 20,000 the nonequilibrium contributions are expected to be much lower, whereas the computational expense of running simulations using this parameter is significantly increased.

**Figure 4:**
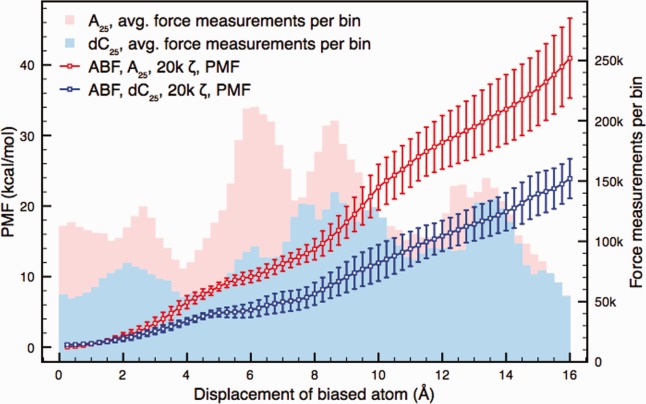
The free energy profiles of A_25_ and dC_25_ translocation through wild type αHL from a set of ABF simulations where the conditions have been set to obtain high quality data, requiring a large computational budget. The reaction coordinate is from the center of the alpha carbons of protein residue 111 at the top of the constriction to 16 Å into the transmembrane barrel from that point. The timestep threshold parameter was 20,000 for these simulations. Each free energy profile was constructed from four samples, the error bars representing the sample-to-sample variation. The histograms represent the number of instantaneous force measurements per bin. The profiles show good agreement with the highly sampled cv-SMD data, exhibiting higher free energy values for A_25_ than dC_25_. The free energy profiles are separated with nonoverlapping error bars after just 3 Å of translocation. At the end of the reaction coordinate, the free energy value of A_25_ translocation is approximately 70% higher than that of dC_25_. [Color figure can be viewed in the online issue, which is available at http://wileyonlinelibrary.com.]

Figure 4 shows free energy profiles from ABF simulations of A_25_ and dC_25_ translocation with a *ζ* value of 20,000. The profiles show good agreement with experimental observations of higher resistance to translocation for poly(A) than for poly(dC). They also exhibit agreement with the highly sampled cv-SMD data, showing consistently higher free energy values for A_25_ than dC_25_. The separation between the free energy profiles is the largest of those represented in this article with nonoverlapping error bars for the vast majority of the reaction coordinate. This figure could also be viewed as representing conditions most similar to those found in experiments, given that the average translocation speed is slower as *ζ* increases, and that the αHL pore in this system is unconstrained. The figure also shows that the error bars are greater for A_25_ than for dC_25_; this finding was also observed in the cv-SMD simulations in Figure 3. As shown by the comparison of *ζ* = 20,000 and *ζ* = 80,000 in Supporting Information, the *ζ* = 20,000 profiles here are still likely to contain some residual nonequilibrium effects. However, at *ζ* values higher than 20,000, the computational expense of producing multisample profiles becomes too great to fully investigate.

Figure 4 also shows the average force measurements per bin from the four samples plotted as a histogram. Here, the average force measurements per bin are generally higher for A_25_ than for dC_25_. It is interesting to note that the comparison of the two profiles could be considered on unequal grounds due to the difference in the amount of sampling between the two and the impact that this has on removing nonequilibrium effects. To remedy this, the value of *ζ* could be increased for dC_25_ or decreased for A_25_; this would make the average force samples per bin more alike across the reaction coordinate. It is also worth noting that doing so would almost certainly improve the separation between the free energy profiles.

### Single nucleotide translocation with an ABF

The comparison of A_1_ and dC_1_ from Figure 3 is revisited here, this time using the ABF methodology instead of cv-SMD. Given the impact of slow-relaxing forces in the polynucleotide chain, it is important to investigate ABF using smaller molecules such as single nucleotides, giving insight into the impact of nonequilibrium effects on the polynucleotide data.

**Figure 5:**
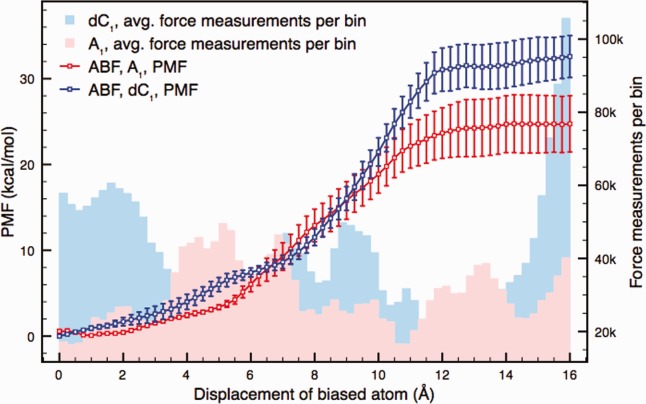
The free energy profiles of A_1_ and dC_1_ translocation through wild type αHL from a set of ABF simulations. The reaction coordinate is from the center of the alpha carbon atoms of protein residue 111 at the top of the constriction to 16 Å into the transmembrane barrel from that point. The timestep threshold parameter was 5000 for these simulations. Each free energy profile was constructed from four samples, the error bars represent the sample to sample variation. The histograms represent the number of instantaneous force measurements per bin. The free energy profiles are separated by nonoverlapping error bars after 10.5 Å of translocation. At the end of the reaction coordinate, the free energy of A_25_ translocation is approximately 33% higher than that of dC_25_. [Color figure can be viewed in the online issue, which is available at http://wileyonlinelibrary.com.]

Figure 5 shows free energy profiles from ABF simulations of A_1_ and dC_1_ translocation with a *ζ* value of 5000. The profiles are constructed from four samples per profile and the error bars represent the sample-to-sample error. As indicated by the data in Supporting Information, a higher value of *ζ* is not as important in reducing nonequilibrium contributions for smaller translocating molecules. The rapid rise in profile gradient after 5 Å and the subsequent leveling out after 11 Å corresponds well to the single nucleotide molecule leaving the confines of the αHL constriction, as was observed in the cv-SMD data. The strong phosphate-lysine interaction found in cv-SMD simulations for dC_1_ is shown to be contributing similarly here as the dC_1_ free energy profile shows a higher cumulative free energy than with A_1_. The histograms showing the average force measurements per bin are largely similar for both nucleotides.

Unlike the cv-SMD profiles of A_1_ and dC_1_ translocation, separation between the two profiles is observed in Figure 5, with dC_1_ showing higher free energy values. It is clear, then, that the greater propensity of dC_1_ to experience a strong electrostatic interaction is observed when using ABF, just as it is when using cv-SMD. Additional samples would be needed to confirm if the dC_1_ electrostatic interaction was experienced to a greater degree with ABF, as is suggested in the figure.

## Comparison of cv-SMD with the ABF Method

Figures 3 and 4 explored the cv-SMD and ABF methodologies for nucleotide translocation through αHL. Both approaches provided qualitative agreement with the experimental finding that A_25_ experiences greater barriers to translocation than dC_25_. This section explores which of the two translocation methods is better suited to explore the nanopore-nucleotide system and the reasons why. First, we compare cv-SMD to ABF based on the general methodological differences; we look at the mode of translocation, consistency with experiment and constraints on the system. We then compare the results from simulations using each methodology in terms of the recreation of experimental conditions and in data quality, the free energy profile shapes, and the free energy profile separation between A_25_ and dC_25_. We also consider the computational efficiency of each approach. The section finishes by extrapolation of our findings to other systems.

### Methodological comparison

SCCR experiments involve the translocation of a polymer through a protein-pore; this is a nonequilibrium process, though it is in a steady-state due to the constant transmembrane potential. This potential drives the polymer through the pore, and the driving force acts on the entire length of the polymer at all times. The free energy landscape of the solvated and ionized molecular system with respect to the translocating molecule, combined with the applied potential, determines the translocation time (a measurable quantity). So, it is this free energy landscape that we wish to estimate using simulation and the difference in translocation time between poly(A) and poly(dC) being a measure that we use to validate our simulations. Therefore, one key point of comparison between cv-SMD/JE and ABF is how closely the methodology matches the experimental process.

In cv-SMD/JE, the molecule is pulled in a nonequilibrium state and, whereas the method causes the molecule to move at constant velocity, the applied force varies in response to the free energy landscape. The driving force is, therefore, different to experiment in this way. Another key difference to experiment is that the driving force is applied to the leading atom of the polymer, whereas experimentally it is applied to the whole molecule. During simulations, pulling a polymeric molecule by its leading end can result in deformation from the equilibrium conformation.^16^ Deformation of the translocating molecule is expected to occur experimentally due to the dimensions of the pore,^7^,^16^ but as a response to the steric hindrance of the constricting pore dimensions, rather than due to being dragged through the solvent. This artifactual form of deformation can be reduced by using a smaller driving force, where relaxation forces have time to act on the molecule. Furthermore, as the reaction coordinate of the ABF methodology is calculated as a function of distance relative to other reference atoms, the free energy profile will be an accurate function of the length of the pore interior, regardless of the movements of the protein. This allows the protein to be completely unconstrained, as discussed in the Subsection “Polynucleotide translocation with an ABF”.

The two methodologies also differ from each other in several other respects. First, the ABF reaction coordinate is one-dimensional (1D) and, therefore, it is not restricted to axes orthogonal to the reaction coordinate; cv-SMD, conversely, is restricted to such orthogonal axes, and so, assuming a stiff spring constant (required in order to use JE), the SMD atom may not stray from a precisely chosen course. In this respect, ABF is closer to experiment than cv-SMD, where under experimental conditions the molecule is free to explore the full internal dimensions of the pore, and the translocation time is a measure of its transmembrane progression (a 1D quantity).

Second, the direction of translocation along the reaction coordinate is not consistent in ABF simulations; therefore, deviations from expected structural conformations of the polynucleotide can vary significantly from sample-to-sample. Such deviations result in a systematic error in the free energy profiles. It is quite straight forward to extrapolate the effect that this has in cv-SMD simulations due to the error being proportional to the consistent pulling speed. With data from several pulling speeds, one could extrapolate what the free energy difference would be at infinitesimally small translocation speed; this is more difficult to do in ABF.

Third, while using cv-SMD/JE requires a balance of statistical and truncation errors in equating the work done to the free energy, ABF involves no such approximations, due to its calculating the free energy directly from the system forces, and applying the biasing force directly into the biased atom's equations of motion.

The ABF methodology, while fundamentally different from cv-SMD in principle and in practice, is nevertheless closely related in certain respects. In ABF for instance, the molecule is permitted to diffuse along the reaction coordinate by a force that is adjusted in response to energetic barriers to translocation. In cv-SMD, as the leading molecule is being forcibly relocated, the actual force applied to it scales in response to the energetic barriers to the relocation, so the process is not in a steady-state in terms of the driving force. In this sense, cv-SMD could be considered more closely related to ABF than it is to constant force-SMD. Additionally, for the polynucleotide-nanopore system, the driving force is applied to a leading residue rather than the whole polymeric molecule, as in cv-SMD simulations. This makes the two methodologies more closely related to each other than they are to methods that use a transmembrane potential as a driving force such as, for example, grid-SMD.^16^

### Data comparison

When analyzing simulation results in relation to experimental results, quantitative comparisons are difficult to draw without key data such as friction coefficients and full pore length translocation data. However, qualitative comparisons may be drawn quite readily. When considering the simulation pulling methods in relation to each other, we may perform a rigorous analysis by drawing comparisons between simulation conditions, error bars, profile shapes, profile separation, free energy values, and computational efficiency.

**Figure 6:**
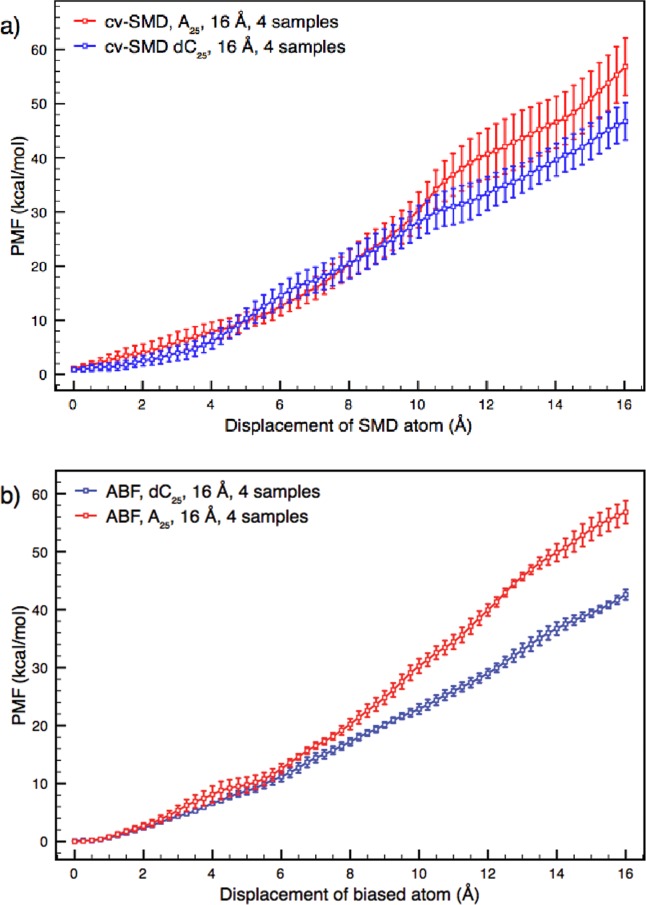
Free energy profiles from cv-SMD (a) and ABF (b) simulations of A_25_ and dC_25_ translocation under comparable conditions. Each profile is calculated from the average of four sample trajectories spanning the full 16 Å reaction coordinate. (a) The ABF parameters were *ζ* = 5000 and bin width 0.25 Å. The free energy profiles are separated with nonoverlapping error bars after 11 Å of translocation. At the end of the reaction coordinate, the free energy value of A_25_ translocation is approximately 45% higher than that of dC_25_. (b) The cv-SMD parameters were a pulling speed of 0.04 Å/ps and bin width 0.25 Å. The free energy profiles are separated with nonoverlapping error bars after 15.5 Å of translocation. At the end of the reaction coordinate, the mean of the free energy values of A_25_ translocation is approximately 20% higher than that of dC_25_. The two methodologies exhibit a greater free energy profile for A_25_ than for dC_25_. Compared to the cv-SMD profiles, the ABF data show greater separation at the end of reaction coordinate; the separation occurs throughout a larger proportion of the reaction coordinate, and the errors are substantially smaller. Taking an average of the error bars over the entire reaction coordinate, the errors in the cv-SMD profiles are approximately 185% larger than the ABF profiles for A_25_, and approximately 270% larger for dC_25_. [Color figure can be viewed in the online issue, which is available at http://wileyonlinelibrary.com.]

A direct comparison of ABF and cv-SMD for the translocation of A_25_ and dC_25_ is shown in Figure 6. Each profile is the average of four sample trajectories, each spanning the full 16 Å reaction coordinate across the pore-constriction. The bin width was set to 0.25 Å and the C_α_ atoms of the αHL pore were constrained in all instances. The ABF parameters were set to *ζ* = 5000 with a bin width of 0.25 Å, whereas the cv-SMD parameters were set to a pulling speed of 0.04 Å/ps. These methodology specific parameters equated roughly to the same average translocation speed. The profiles show that both methodologies exhibit higher free energy values for A_25_ than for dC_25_. Additionally, the mean free energy values at the end of the reaction coordinate for ABF are within error bars for cv-SMD for the same polynucleotide.

Figure 6 also shows that use of each methodology leads to notable differences. The ABF method manifests a greater separation between the free energy profiles of A_25_ and dC_25_ by the end of the reaction coordinate. At the end of the reaction coordinate, the free energy value of A_25_ translocation is approximately 20% higher than that of dC_25_ when cv-SMD is used. The difference is approximately 33% when ABF is used. The separation is also aided by the considerably smaller error bars in the ABF profiles. By contrast, the error bars on the A_25_ and dC_25_ free energy profiles can be seen to overlap in the case of cv-SMD for the majority of the reaction coordinate. The separation between A_25_ and dC_25_ at the end of the reaction coordinate is 15.8 ± 4.9 kcal/mol in ABF and 10.1 ± 8.8 kcal/mol using cv-SMD, therefore, the separation of the profile means is larger in addition to having smaller errors using ABF. Taking an average of all the free energy profile error bars across the reaction coordinate, the average errors in the cv-SMD profiles are approximately 185% larger than with ABF for A_25_, and approximately 270% larger for dC_25_.

The error bars are observed to be smaller when using ABF for a couple of reasons. First, as discussed, the binning error is negligible due the large number of measurements taken per bin, improving the statistical quality of the calculations. Second, single samples of ABF, with its translocative motion determined largely by self-diffusion and not by being forced along the reaction coordinate, may be more representative of the true free energy landscape, and so the sample-to-sample fluctuations are lower. Consider that an infinitesimally slowly moving molecule is likely to sample all accessible phase space configurations and energy values to a degree which is fully representative of the free energy landscape, and therefore, multiple samples of infinitesimally slowly moving trajectories will have zero sample to sample free energy profile fluctuation. Equally, a fast moving entity will sample less of the accessible phase space; therefore, more samples will be required to construct a meaningful free energy profile. It follows, then, that a methodology which samples the phase space more effectively will represent the free energy landscape better per sample, and so the sample-to-sample variation will be reduced. It is likely that the lack of constraints along axes orthogonal to the reaction coordinate also contributes to this effect.

### Computational efficiency

To fully compare each method, one must also look at the computational cost under comparable conditions, in addition to the quality of the output. In general there is roughly a 3.5% increase in computation time for an ABF simulation compared to a cv-SMD simulation for a fixed number of timesteps with the same number of cores on the same system (tested on the XSEDE machine Kraken at 576 processors). This is because an ABF simulation must perform additional calculations for the generalized coordinates of the biased and reference atoms, and calculate the average instantaneous force acting on the biased atom. Calculations based on the cv-SMD harmonic spring and the position of the reference atom are comparatively simple, and therefore, less computationally demanding.

For the ABF simulations that give rise to the profiles in Figure 6, the bin width (0.25 Å) and *ζ* value (5000) lead to roughly 2 million timesteps per sample trajectory at a total cost of roughly 25,000 CPU hours for a four sample profile. Here, each sample trajectory is produced from two or more simulations in blocks of 100,000 to 1 million timesteps per simulation until the full reaction coordinate is sampled. With a cv-SMD pulling speed of 0.04 Å/ps, for a 16 Å translocation, 2.4 million timesteps are required per sample at a total cost of 29,000 CPU hours for a four sample ensemble average. Here, each sample is produced from four simulations, the combined simulations covering the full reaction coordinate. There is additional computational time required in cv-SMD simulations under the conditions we have used in order to produce the reaction coordinate segment overlap; the explanation for this is provided by Martin et al.^31^

It should be noted that a relatively consistent progression along the reaction coordinate for the ABF simulations under these conditions is aided by undesirable slow nonequilibrium relaxational effects. With smaller translocating molecules, or higher *ζ* values to allow more time for the conformations to relax (thus producing a more correct profile), the number of timesteps required to sample the whole trajectory would increase and be difficult to predict. As shown in Supporting Information, where *ζ* = 80,000, sampling the reaction coordinate requires roughly 16 million timesteps for a polymeric chain and 20 million timesteps for a single nucleotide. In cv-SMD simulations, the quality of the data may also be improved by slowing down the translocation. In the case of cv-SMD, the increase in computational cost is precise, and therefore, straightforward to plan and manage.

For the conditions given for this comparison, ABF displays numerous advantages; it possesses fewer sources of error, smaller errors, better separation of free energy profiles, lower computational cost, fewer constraints, and greater degrees of freedom in axes orthogonal to the reaction coordinate.

### Extrapolating to other systems

The question remains as to whether this comparison would hold up in other systems/conditions. To answer this, we must consider individual contributions to each free energy profile. In cv-SMD/JE, there are two sources of error from the implementation of the methodology: the harmonic spring and the truncation of the cumulative term in the use of Jarzynski's identity. The latter will have a contribution in other systems, regardless of size or pulling speed. The harmonic spring leads to an increase in the statistical noise of the output as the harmonic spring constant is increased, yet it must be high enough to approximate a stiff spring. For larger translocating molecules, the spring constant must be scaled up to continue approximating a stiff spring, hence it becomes necessary to introduce more statistical noise. The higher statistical noise will increase the binning error in the free energy profiles. Therefore, the cv-SMD error would be expected to increase for larger translocating molecules. This scaling of binning error may also be affected by the pulling speed, where faster pulling speeds require higher spring constants in order to approximate a stiff spring, thereby increasing the error contribution.

Even if the binning error were completely negated in the cv-SMD profiles, the sample-to-sample contributions to the errors are larger than those of the ABF profiles. This may appear surprising as, for the ABF simulations, the reaction coordinate is not restrained in axes orthogonal to it. This lack of restraint increases accessible regions of the phase space, which one would expect to increase the sample-to-sample fluctuations. The converse is in fact observed, where each sample appears to represent the free energy landscape well, resulting in low sample-to-sample fluctuations. It is possible that the constrained reaction coordinate in the cv-SMD case imposes certain conformations on the translocating molecule, to a degree which may not be proportionally representative of the ensemble phase space, thereby resulting in more varied individual samples. It is, moreover, feasible that the sampling of phase space is also improved by the translocative motion in ABF simulations being determined largely by self-diffusion rather than rigidly implemented relocation, again leading to lower sample-to-sample fluctuation. For these advantages in the ABF sampling to be allowed to flourish, the translocating molecule must be permitted sufficient time within each bin along the reaction coordinate, whereas the time spent in each bin would be reduced if the average translocation speed were increased. Therefore, at higher speeds, one might expect the sample-to-sample fluctuations to occur to a similar degree in both methodologies, whereas at slower speeds, the ABF methodology would produce better data for a given computational budget. Further investigation would be required to fully answer the question as to how the ABF and cv-SMD methodologies compare in other systems and/or conditions; it is nonetheless clear that, for the translocation of polynucleotides through the αHL protein pore, ABF stands out as the methodology of choice.

## Conclusions

We have conducted a thorough comparison of cv-SMD with ABFs for the translocation of nucleic acid molecules through the αHL protein pore. ABF was used to translocate polymers through αHL for the first time, while existing investigations of this type using cv-SMD were enhanced. The resulting free energy profiles from ABF translocation were within error bars of those from cv-SMD translocation and showed that A_25_ experienced greater barriers to translocation than dC_25_. However, using ABF, the error bars were found to be notably smaller and the separation between the free energy profiles of A_25_ and dC_25_ translocation was larger. Given that ABF presents these advantages in the statistical quality of the data, as well as other advantages intrinsic to the methodology (freedom to explore the internal dimensions of the pore, introduction of fewer errors in the calculation of free energy), and under our conditions is less computationally intensive for obtaining similar results to cv-SMD, we find that ABF method is a natural choice for future work of this type. It should be noted that cv-SMD retains a notable advantage over ABF in that it has a set number of timesteps required to traverse a reaction coordinate distance, allowing precise planning of simulation time and a computational budget.

With ABF established as the preferred method, future investigations could aim to compare ABF to alternative translocation methods, particularly metadynamics and/or grid-SMD. With Oxford Nanopore Technologies making progress in the field of nanopore sequencing, it would also be of great interest to reconstruct their most successful αHL nanopores in simulations that harness such translocation methods. The insight gained could be used to improve the experimental system while the race for cheaper and faster sequencing technologies goes on.
