# Supplementary material for: Comparative analysis of nucleotide translocation through protein nanopores using steered molecular dynamics and an adaptive biasing force
Source: J Comput Chem. 2014 Jan 9;35(9):692–702. doi: 10.1002/jcc.23525 (PMC4274958; doi:10.1002/jcc.23525)
Supplement: Supplementary file 1 — Additional Supporting Information may be found in the online version of this article. [file jcc0035-0692-SD1.pdf]

Supporting Information for Comparative Analysis of Nucleotide Translocation through Protein Nanopores using Steered Molecular Dynamics and an Adaptive Biasing Force

Hugh S. C. Martin,* Shantenu Jha,† Peter V. Coveney‡

November 8, 2013

1 Theoretical Background

In this section we provide an account of the theoretical background to the cv-SMD/JE and ABF translocation methodologies and their associated means of determining the free energy of translocation.

1.1 Constant Velocity-Steered Molecular Dynamics

SMD provides a means of exploring longer translocation trajectories on a smaller timescale while retaining atomistic detail. This method is inherently non-equilibrium in nature. Such non-equilibrium approaches are often used instead of reducing the detail of the model to a coarse-grained one in order to increase the scope of the investigation. MD programs such as NAMD[1] allow simulated processes to be steered by introducing non-equilibrium forces. In SMD, an atom can have a directional force applied to it, causing the atoms (and any other particles coupled to them) to move with the direction of the force. The consequence of this applied force is that a significant degree of movement can be induced in a relatively short timeframe, and the full atomistic dynamics of the process can be examined.

By applying SMD to threading nucleic acid sequences through nanopores, the translocation process can be replicated in an atomistically detailed model within a timeframe that is feasible to simulate. By pulling the nucleic acid strand at constant velocity (known as constant velocity steered MD or cv- SMD[1]), a translocation of known distance can be performed. In cv-SMD, an atom or the centre of a group of atoms is harmonically restrained to a point in space that is shifted in the chosen direction. The harmonic restraint can be thought of as a spring attached to a dummy atom, the strength of the restraint being given

*Centre for Computational Science and Department of Chemistry, UCL, 20 Gordon Street, London, UK
†Department of Electrical Engineering Building, Rutgers, 94 Brett Road, Piscataway, New Jersey, USA
‡Centre for Computational Science and Department of Chemistry, UCL, 20 Gordon Street, London, UK

by a force constant k. The simulation outputs the force in pico-newtons (pN) experienced by the spring in the direction of pulling (the reaction coordinate).

We want to convert the force output of the simulation to the free energy of translocation, giving free energy profiles which can tell us about energetic barriers to translocation. Free energy, however, is an equilibrium property while cv-SMD is a non-equilibrium technique. To overcome this, Jarzynski's Equality[2] can be used. JE equates equilibrium free energy to the ensemble average of the exponential work from a non-equilibrium process. The work can be calculated from the force output using a force-distance integral.

Consider a process changing a parameter, λ, of a system at an initial equilibrium state at time zero, to a final state at time t. The second law of thermodynamics states that the average work done during a process, $\langle W \rangle$, on the system cannot be smaller than the Helmholtz free-energy difference between the initial and final states of λ:

$$\Delta F = F(\lambda_t) - F(\lambda_0) \leq \langle W \rangle \tag{1}$$

Here, and subsequently, angular brackets $\langle \cdots \rangle$ imply an ensemble average in the statistical mechanical sense. Jarzynski determined that the free-energy difference between the two states can be related to the work, W, by the following equality:

$$e^{-\beta \Delta F} = \left\langle e^{-\beta W} \right\rangle \tag{2}$$

Here, β is the inverse temperature, $(1/k_B T)$, where k_B is Boltzmann's constant.

1.2 Adaptive Biasing Force

Adaptive biasing force is an advanced form of umbrella sampling (US) which estimates the free energy landscape during the sampling simulation and applies biasing forces which allow for effective sampling. The algorithm used to determine the adaptive biasing force was developed by Darve and Pohorille [3,4] and implemented within the NAMD code by Hénin and Chipot[5].

In US, an external potential is applied to allow for the exploration of higher energy configurations and the states that they separate. Here, the free energy along a chosen reaction coordinate (ξ) is given by:

$$F(\xi) = \frac{1}{\beta} ln \mathcal{P}_\xi - \mathcal{U}_{bias} + F_0 \tag{3}$$

where $F(\xi)$ is the free energy of the state at a particular value of ξ; $\mathcal{P}_\xi$ is the probability density of finding the system at ξ; $\mathcal{U}_{bias}$ the applied external potential; β the inverse temperature, and F_0 is a constant. If $\mathcal{U}_{bias}$ is tuned to be the exact opposite of the free energy, $-F(\xi)$, then the free energy landscape will effectively be flattened out. This then permits uniform sampling along the reaction coordinate. Knowing the landscape of $-F(\xi)$ implies an *a priori* knowledge of the free energy landscape. If the $\mathcal{U}_{bias}$ deviates from $-F(\xi)$ then the reaction coordinate will be poorly sampled. Thus, when using US for unfamiliar systems, good sampling of the reaction coordinate is very difficult to realise.

Adaptive biasing force (ABF) is a method of reaction coordinate sampling which applies a continuous biasing force, which is tuned during the simulation to the cumulative estimate of

the free energy landscape. Uniform sampling can thus be achieved by on-the-fly calculation of $F(\xi)$ and the implementation of this information in the form of an external bias. The implementation of ABF, therefore, requires no knowledge of the free energy landscape prior to the simulation.

The free energy in an ABF simulation can be calculated from the forces acting on the biased atom. The derivative of the free energy of translocation with respect to the reaction coordinate can be expressed in terms of configurational averages at constant ξ:

$$\frac{dF(\xi)}{d\xi} = \left\langle \frac{\partial \mathcal{V}(x)}{\partial \xi} - \frac{1}{\beta}\frac{\partial ln|J|}{\partial \xi} \right\rangle_{\xi} = -\langle f_{\xi} \rangle_{\xi}\,. \tag{4}$$

The $\partial \mathcal{V}(x)/\partial \xi$ term represents the physical forces exerted on the system, as derived from the potential energy function, $\mathcal{V}(x)$. The $(1/\beta)(\partial ln|J|/\partial \xi)$ term represents a geometric correction where $|J|$ is the determinant of the Jacobian for the transformation from generalised to Cartesian coordinates; it accounts for the difference in phase space availability as the reaction coordinate varies. $\langle f_{\xi} \rangle_{\xi}$ is the average force acting along reaction coordinate ξ, derived from instantaneous force components f_{ξ}.

The biasing force of the ABF method is calculated as the negative of the average force acting along reaction coordinate ($\langle f_{\xi} \rangle_{\xi}$) derived from instantaneous force components (f_{ξ}). For its practical implementation, f_{ξ} is accumulated in small bins along the reaction coordinate ($\Delta\xi$) to provide an estimate of the change in free energy with respect to the reaction coordinate, $dF(\xi)/d\xi$. The adaptive biasing force, $\mathbf{f}^{ABF}$, is thus defined as:

$$\mathbf{f}^{ABF} = \frac{dF(\xi)}{d\xi} = -\langle f_{\xi} \rangle_{\xi}\,, \tag{5}$$

where $F(\xi)$ is the current estimate of the free energy as a function of the reaction coordinate. The calculated biasing force, $\mathbf{f}^{ABF}$, is introduced into the equations of motion during a simulation. With the biasing force applied, the overall forces acting on the biased atom(s) along the reaction coordinate within a bin average to zero over time. This is because the biasing force approximately opposes the energetic barriers to translocation. As the applied biasing force roughly matches the free energy landscape, evolution along the reaction coordinate is largely governed by a process of self-diffusion.

The instantaneous force, f_{ξ}, fluctuates to a high degree; thus the average force will initially take inaccurate and physically meaningless values. Therefore, the biasing force is not implemented within a particular bin until a threshold of force measurements has been accumulated; the biasing force is then introduced gradually via a linear ramp. The threshold value, ζ, is an input parameter in units of timesteps and its optimal value is dependent on the nature of the system. A higher value of ζ will help in the reduction or removal of excessive non-equilibrium effects; therefore, it will need to be larger for systems with slowly relaxing degrees of freedom, for example, those systems with large, flexible translocating molecules. ζ heavily influences the translocation/simulation time, though it is not the only contributing factor due to the dependence of the motion of the biased atom(s) on self-diffusion. This process is described in Figure 1.

To keep the atom(s) within the bounds of the reaction coordinate, harmonic boundary forces are applied at either end. The position of the boundaries and the value of their

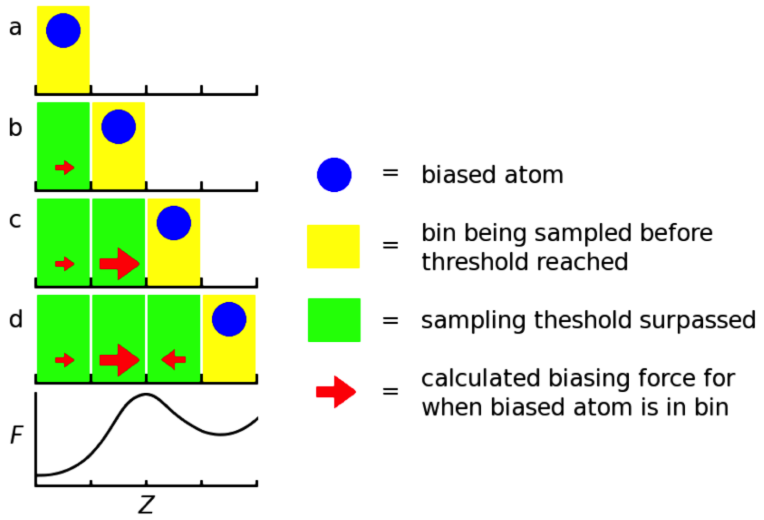

Figure 1: Illustration of the ABF methodology. The horizontal axes represent the translocation reaction coordinate, which is the distance along the pore. The reaction coordinate is discretised into bins of equal size. The ABF methodology can be summarised through the time sequence represented by illustrations (a)-(d) and the free energy profile at the bottom. (a) The biased atom (blue circle) is within the first bin of the reaction coordinate. The biasing force is being estimated (yellow rectangle). (b) The biased atom has been sampled more than the threshold, ζ, in the first bin and the biasing force (red arrow) has allowed the atom to diffuse into the subsequent bin. (c) The biased atom has been sampled more than the threshold ζ in the second bin and has diffused into the subsequent bin. The biasing force to move the atom from the second bin was larger than in the first, due to the free energy landscape. (d) The biased atom has reached the end of the reaction coordinate; it may continue to diffuse backwards across the reaction coordinate if the simulation continues. In this illustration, the atom has tended to move forward at each stage, but it may also diffuse in the reverse direction at any time, since the biasing force is intended to match rather than exceed the free energy barriers.

force constants are selected as simulation input parameters. The boundary forces are rigidly implemented and may influence the biased atom when it is near to them[6], therefore it is beneficial in this respect to ensure that the distance between the boundaries is as long as possible. However, as we shall see later in this paper, there are benefits to segmenting the full reaction coordinate into several shorter intervals.

The ABF reaction coordinate is often defined in terms of relative atomic positions, that is, the distance between selected atoms or groups of atoms. In the latest implementation of ABF in NAMD, the reaction coordinate may be defined in various ways, including bond angles, radii, and RMSD values[7]. For the studies in this paper, the reaction coordinate is most appropriately designated in terms of inter-atomic distances in the z-axis direction. Since the reaction coordinate is relative to two atoms or groups of atoms, the Cartesian coordinates of the moving and reference atoms or groups must be converted into generalised coordinates.

By imposing a biasing force on the molecule of interest, and simultaneously allowing the free energy of translocation to be calculated, ABF presents itself as an ideal candidate for the nucleotide-nanopore translocation system.

2 Snapshots of Translocation Conformations

Figures 2 and 3 show snapshots representing the translocation simulations in this paper. Figure 2 shows dC_{25} translocation over a distance of 48 Å using cv-SMD; the image is also a useful reference for the ABF simulations in this paper, and those concerned with translocating A_{25}. Figure 3 shows dC_1 translocation over 16 Å using ABF; the image is also a useful reference for the cv-SMD simulations in this paper, and those concerned with translocating A_1.

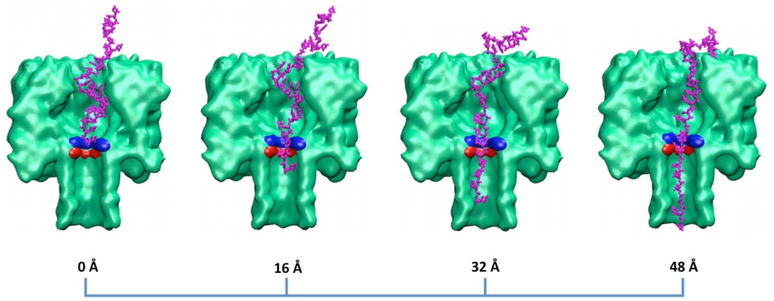

Figure 2: Snapshots of dC_{25} translocation for 48 Å using cv-SMD. The image provides an indication of the translocation lengths and conformations of single stranded nucleic acids reported in this paper. Residues 147 and 113 are marked in blue and red respectively to indicate the position of the pore constriction.

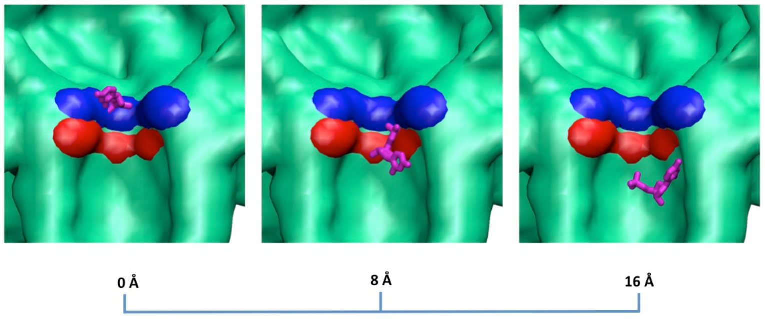

Figure 3: Snapshots of dC_1 translocation for 16 Å using ABF. The image provides an indication of the translocation lengths and conformations in this paper. Residues 147 and 113 are marked in blue and red respectively to indicate the position of the pore constriction.

3 Exploration of Constraints on the Nanopore

In this section we explore the interesting option of leaving the protein completely unconstrained, which is permitted due to the nature of the calculation of the reaction coordinate in ABF simulations.

Before we can examine ABF-induced polynucleotide translocation in detail, it is important to recall that the reaction coordinate of the ABF methodology is calculated as a function of distance from the translocating molecule to a reference atom or set of atoms. In cv-SMD, the reaction coordinate is a function of the Cartesian coordinates of the system. If the protein were to move during the simulation, the free energy profile would no longer be an accurate function of the pore interior; therefore pin-pointing the free energy profile to specific pore residues would not be possible. This necessitates the constraining of the αHL pore in cv-SMD simulations. For ABF simulations, if the reference atoms are reasonably close to the translocating molecule, the free energy profile will be an accurate function of the length of the pore interior, regardless of the movement of the protein. This aspect of ABF provides an opportunity to leave the protein completely unconstrained, allowing the simulation to further approach conditions found in experiment.

Leaving the αHL protein unconstrained could have important consequences. In a comprehensive study of the αHL pore in MD simulations, the transmembrane barrel was found, on average, to be elliptical in shape[8]. The study found that the pore adopted seven different orientations of this shape, alternating between them on the scale of several nanoseconds. Constraining the protein prevents this kind of activity, and the consequences this might have on the simulations performed in this paper are unknown. Therefore, avoiding the constraining of the protein is could be important for accuracy.

Figure 4 shows free energy profiles from ABF simulations of A_{25} and dC_{25} translocation through an unconstrained (Figure 4(a)) and constrained (Figure 4(b)) αHL pore, both with a ζ value of 5000. Each profile is constructed from four samples and the error bars represent the sample-to-sample fluctuations. At the end of the 16 Å reaction coordinate in both cases, there is a separation of the A_{25} and dC_{25} profiles with non-overlapping error bars, with A_{25} showing a higher cumulative free energy of translocation. The figure also shows the average number of force measurements per bin from the four samples, in the form of histograms.

While the simulations of the unconstrained and constrained systems give similar results in terms of their end-points (Figures 4(a) and 4(b) respectively), they also differ in some respects. Firstly, the histograms representing the instantaneous force measurements per bin (plotted against the right-hand y-axes) for the translocation of both polynucleotides have, on average, higher values in the unconstrained protein system, showing that the simulations need to be run for more timesteps in order to sample the full reaction coordinate. This is likely a consequence of the protein being allowed to shift position, to a degree in response to the translocating polynucleotide. Such shifts in the protein position appear to have a minimal impact on the total free energy difference across the reaction coordinate; as Figure 4 shows, the profile end points overlap to within errors when comparing the same translocating molecule (for example, comparing the A_{25} profiles in constrained and unconstrained protein conditions).

Secondly, the free energy error bars are larger with the unconstrained protein. Taking an average of the size of the error bars across the whole reaction coordinate, the errors are

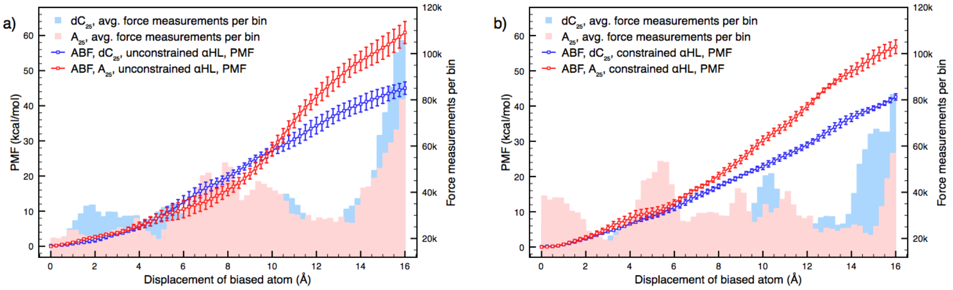

Figure 4: Free energy profiles of A_{25} and dC_{25} translocation through wild type αHL from a set of ABF simulations with the protein constrained and unconstrained. The reaction coordinate is from the centre of the alpha carbon atoms of protein residue 111 at the top of the constriction to 16 Å into the transmembrane barrel from that point. The timestep threshold parameter was set to 5,000 for these simulations. Each free energy profile was constructed from four samples, the error bars representing the sample-to-sample variation. The histograms represent the number of instantaneous force measurements per bin. (a) Free energy profiles from simulations with the αHL pore completely unconstrained. The free energy profiles are separated with non-overlapping error bars after 11 Å of translocation. At the end of the reaction coordinate, the free energy values of A_{25} translocation are approximately 45% higher than those of dC_{25}. (b) Free energy profiles from simulations with the C_α atoms of αHL constrained. The free energy profiles are separated with non-overlapping error bars after 6 Å of translocation. At the end of the reaction coordinate, the free energy values of A_{25} translocation are approximately 33% higher than those of dC_{25}. Taking an average of the error bars across the whole reaction coordinate, the errors are approximately 70% larger when unconstrained for A_{25}, and approximately 125% larger when unconstrained for dC_{25}. The A_{25} profile exhibits a curved shape in the unconstrained system, and a more uniform gradient in the constrained system.

approximately 70% larger unconstrained compared to constrained for A_{25} translocation, and approximately 125% larger for dC_{25} translocation. When the protein is unconstrained, the scope of the phase space that can be explored becomes greater, therefore the sample-to-sample fluctuations are expected to be larger for a finite amount of sampling.

Thirdly, the separation of the A_{25} and dC_{25} profiles with non-overlapping error bars begins at different points in the constrained system (6 Å) compared to the unconstrained one (11 Å). The shapes of the profiles also play a role in this observation; the A_{25} profile exhibits a curved shape in the unconstrained system, while this is absent from the constrained system. Given the expected impact of the phosphate-lysine interaction as previously demonstrated, the absence of curves in the profiles could be interpreted as a loss of detail; however, the presence of curves in the free energy profiles from the unconstrained system may be due to the shifting position of the protein, thus being a consequence of the force from the translocating molecule, this would be an undesirable effect.

Finally, the separation of the A_{25} and dC_{25} profiles is larger for the unconstrained system than for the constrained system. At the end of the reaction coordinate, the cumulative

free energy value of A_{25} translocation is approximately 45% higher than that of dC_{25} for the unconstrained system, and approximately 33% higher in the constrained system. This difference can be attributed to error, however, as the profile end-points lie within error bars of each other.

In deciding whether to constrain the protein or not, one must consider the impact of a shifting protein, both in terms of conformation and overall position. Based on the results in Figure 4, the differences in the data give rise to a trade-off between the size of the errors and the accurate re-creation of experimental conditions. For the αHL -polynucleotide system at least, the choice does not dramatically impact the end result of the free energy profiles.

4 The Influence of Force Measurements Per Bin on ABF Trajectory Progression

In Adaptive Biasing Force (ABF) translocation simulations, choosing a value for the parameter 'force measurements per bin' (ζ) has a direct effect on the time that the biased atom can spend in a bin along the reaction coordinate before a biasing force is applied. A higher ζ ensures a longer simulation and a slower average translocation speed. The nature of ABF means that the instantaneous translocation speed is variable, thus the average speed across the full reaction coordinate may be considered as an indicator of the impact of non-equilibrium effects. The average translocation speed is ideally kept to a minimum to ensure that non-equilibrium effects, and their impact on the free energy estimate, are kept low. A low average translocation speed can also lead to overly long simulation times, therefore a balance between speed and computational expense is required within a given computational resource budget.

The impact that the ζ parameter has on the biased atom's progression along the reaction coordinate is shown in 5. The data is produced from simulations of polynucleotide (dC_{25}) translocation across a 16 Å trajectory in α-hemolysin (αHL). The figure shows the displacement of the biased atom plotted against the number of timesteps simulated at ζ values of 0, 5000, 20000 and 80000. At ζ=0, the translocation of the biased atom is extremely rapid; at ζ=5000 about 2 million timesteps are required in order for the biased atom to translocate the full length of the reaction coordinate; at ζ=20000 about 5 million timesteps are required; at ζ=80000 about 16 million timesteps are required. The figure indicates that the time taken for the biased atom to traverse the reaction coordinate shows a strong dependance on the value of ζ, though it is not directly proportional, the figure show that increasing ζ 4-fold results in less than a 4-fold increase in translocation time. The fact that the increase in translocation time is not directly proportional to the value of ζ is expected, since the value of ζ only directly affects the parts of the simulation when the biased atom does not yet have a biasing force applied to it. The value of ζ may influence the value of the applied bias due to its impact on reducing non-equilibrium effects, but this effect is unlikely to be directly proportional to the ζ value.

5 also shows that the fluctuations in reaction coordinate progression increase with a higher value of ζ. The degree of fluctuation in translocation progression is similar when ζ=5000 and ζ=20000. At ζ=80000, the translocation progression fluctuates to a significant degree. This

relation of degree of fluctuation as a result of ζ is clear even when the x-axis is normalised so that the x-axis end points are the same for each value of ζ (not shown). It should be noted that high fluctuations in the translocation progression can be viewed as evidence that the biasing force is accurately opposing the free energy barriers. This is because the ABF methodology does not aim to exceed barriers to translocation, but rather match them in order for self-diffusion to dictate translocation. In this way, translocation is permitted, rather than induced, meaning that with barriers effectively removed, translocation is likely to occur in both directions along the reaction coordinate. Only if these energy barriers are exceeded would translocation be expected to be unidirectional.

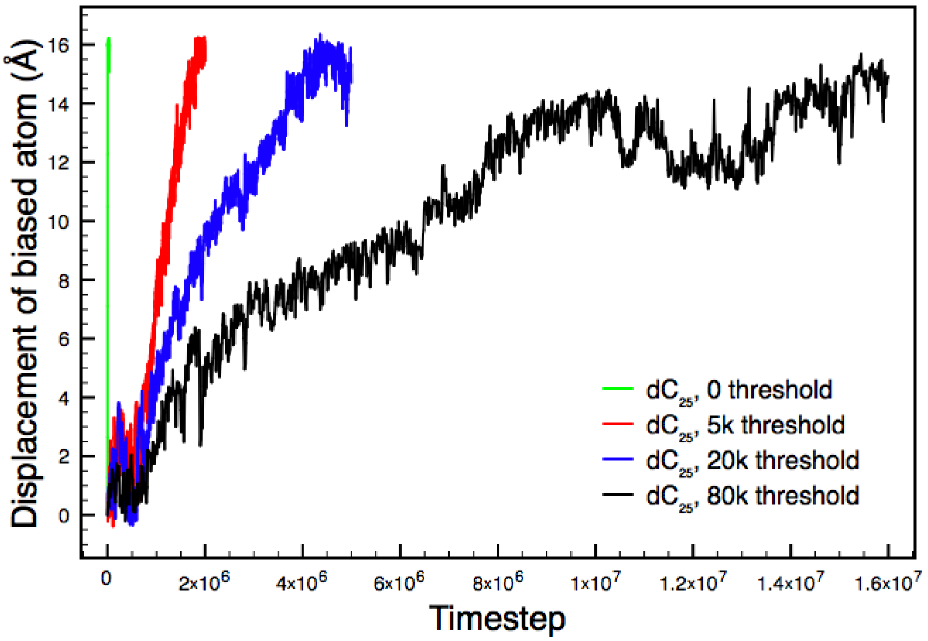

Figure 5: The z-axis displacement of the biased atom plotted against the simulation timestep in dC_{25} ABF simulations with ζ values of 0, 5000, 20000 and 80000 ζ. The time taken to traverse the reaction coordinate shows a strong dependance on the value of ζ. The tendency for the biased atom to move forward is seen to become less consistent at ζ=80000. The sample-to-sample variation in translocation time increased with higher ζ values, since a higher ζ value results in a greater dependance of the translocation on self-diffusion. The single samples represented here are sufficient to illustrate the effect of increasing ζ.

The size of the translocating molecule has a significant influence on its progression along the reaction coordinate. 6 shows the displacement of the biased atom plotted against the number of timesteps simulated at ζ=80000 for dC_1 and dC_{25} translocation simulations. The progression along the reaction coordinate is seen to fluctuate to a greater degree in the case of dC_1, resulting in a roughly 25% longer time to traverse the entire reaction coordinate. This trend can be explained on a molecular level; the smaller dC_1 molecule possesses greater freedom to diffuse in both directions along the reaction coordinate due to the size of the molecule. The biased atom of the polynucleotide molecule also has the whole chain to

contend with, if it is to move in the reverse direction.

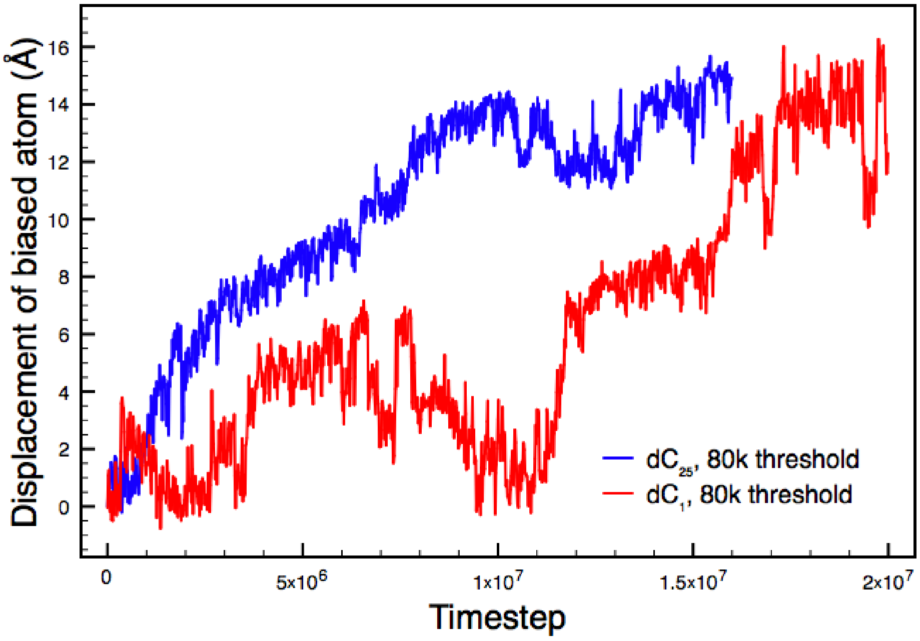

Figure 6: The z-axis displacement of the biased atom plotted against the simulation timestep in dC_{25} and dC_1 ABF simulations with a ζ values of 80000. The polynucleotide is shown to have a much greater tendency to progress forwards along the reaction coordinate. The single nucleotide more readily moves in the reverse direction, displaying more freedom of movement or a greater dependence of translocation on self-diffusion. This typically resulted in a longer translocation time for single nucleotides compared to polynucleotides, as illustrated in this example.

The influence of the size of the translocating molecule on progression along the reaction coordinate may also be related to the methodological implementation of ABF; consider the leading atom of the polynucleotide being encouraged to move along the reaction coordinate by the biasing force. As the biased atom moves, the polymeric equilibrium conformation is deformed. The altered conformation of the polynucleotide may begin to relax once the leading atom slows or stops its progression. For example, when the atom moves into a new bin along the reaction coordinate, thus the biasing force is not yet being applied in that new bin. At all times during states of perturbation and relaxation of the translocating molecule, the instantaneous force acting on the biased atom is measured and used to calculate or improve the biasing force. As the biased atom moves into unexplored bins along the reaction coordinate, the system will evolve from a more equilibrated conformation to a more perturbed one. A freshly perturbed polymeric chain will resist the change by pulling back on the leading atom, giving an increased average instantaneous force in the reverse direction, so the calculated biasing force will be higher in the forward direction. Therefore, as the chain then begins to relax and the pull from the chain reduces, the biasing force will be disproportionately large in the forward direction due to the average instantaneous force for

that bin having been largely calculated from the more greatly perturbed state. This effect will encourage consistent progression in the forward direction along the reaction coordinate, since the energetic barriers to translocation are being exceeded by the biasing force, rather than being matched by it.

The phenomenon of translocation progression being influenced by molecular conformation will likely be a factor in all multi-atom molecules, with larger and/or more flexible molecules exhibiting it to a greater degree, resulting in shorter translocation times. It should be noted that this effect results in a deviation from the free energy profile which accurately reflects the free energy landscape; thus it is a systematic error that increases with more slowly relaxing molecules, requiring a larger ζ value to reduce the impact of this effect.

5 Influence of Force Measurements Per Bin on Free Energy Profiles

The impact that the force measurement threshold parameter has on the translocation free energy profiles is explored in this section. It is important to determine the non-equilibrium contributions to the free energy estimate, as such contributions can represent deviation from physically meaningful values.

In the absence of the force threshold, the free energy profiles are dominated by non-equilibrium effects resulting from rapid translocation. 7 shows the relative heights of single sample free energy profiles from ABF simulations dC_{25} translocation at ζ=0, 5000, 20000 and 80000. The figure shows the considerable difference between the free energy profile when ζ=0 and the profiles at the other three values, exhibiting a 20- to 50-fold increase in the cumulative free energy value after 16 Å of translocation. As discussed in the previous section, the difference arises due to the slowly relaxing conformations of the polynucleotide, which, as this figure shows, have a large impact on the cumulative value of the free energy profile.

Low values of ζ also alter the shape of the free energy profiles. 8 shows single sample free energy profiles from ABF simulations of A_{25} and dC_{25} translocation at ζ=0 and ζ=20000. The free energy profiles have been normalised so that the y-axis end points are the same for each profile to illustrate the difference in the profile shapes. The curved shape present when ζ=20000 is entirely absent at ζ=0. This curve is attributed to the influence of the constriction of αHL due to the correlation of the high gradient portions corresponding to molecular configurations of high steric hindrance and electrostatic interactions; therefore it can be inferred that the ζ=0 profile is dominated by non-equilibrium effects, highlighting the importance of a carefully selected ζ value.

9 shows free energy profiles from single sample ABF simulations of dC_{25} translocation at ζ values of 5000, 20000 and 80000, plotted with histograms of the force measurements per bin used to calculate the biasing force in that bin. The high number of measurements found at the end of some of the force measurement histograms is a result of the ABF algorithm, which continues to take force measurements after the reaction coordinate has been fully sampled. A fully sampled reaction coordinate will have a free energy estimate for every bin along the length of the reaction coordinate, estimated from a number of instantaneous force

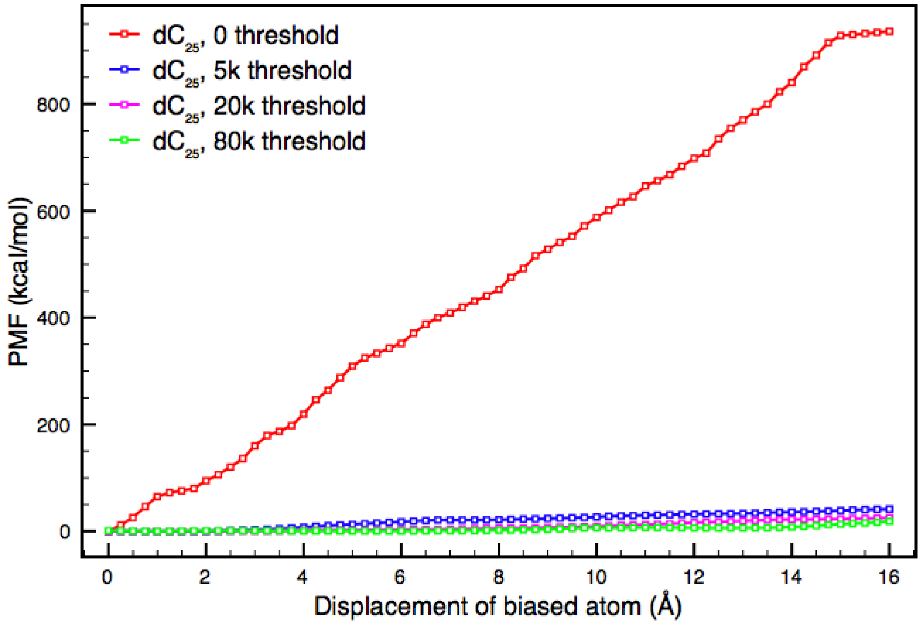

Figure 7: Free energy profiles from ABF simulations of dC_{25} translocation comparing ζ values of 0, 5000, 20000 and 80000. At higher ζ values, slow relaxing configurations are given more time to relax, resulting in a reduced cumulative free energy value. At a ζ value of 0, the free energy profile is high, whereas the profiles from simulations with 5000, 20000 and 80000 ζ begin to converge within a close region.

measurements greater than the value of ζ. Since the number of timesteps required to fully sample the reaction coordinate is unknown, one must add blocks of timesteps simulated until the reaction coordinate has been sampled; this can lead to additional time spent sampling the end of the reaction coordinate, as observed in these profiles. Variation in the force measurements per bin along the reaction coordinate occur to a degree, the maximum peaks in the histograms tend to be higher for larger ζ values, in a manner which is roughly proportional to ζ. Peaks in the force measurements per bin histograms occur with little regularity in terms of peak height and position. While the variation in sampling does not have a dramatic impact on the shape of the free energy profiles, the additional sampling at the end of the reaction coordinate for ζ=5000 and 20000 in 9 correlates with a levelling off of the free energy profiles.

As shown in 7 and 9, the cumulative free energy values begin to converge as ζ increases. The cumulative value where ζ=80000 is assumed to have the lowest systematic error in terms of slowly relaxing conformations. However, at ζ=80000, the shape of the profile exhibits a strong dependance on the sampling per bin. By cross comparison with 5, the plateau in the free energy profile between 10 and 14 Å in 7 is seen to correspond to a high degree of variance in translocation position in 5. It is difficult to determine the precise nature of this effect without more samples to compare; due to the great computational expense of performing simulations at ζ=80000, this has not been performed for this study. For the translocation of polymers using ABF, it may be possible to extrapolate the impact that this systematic error

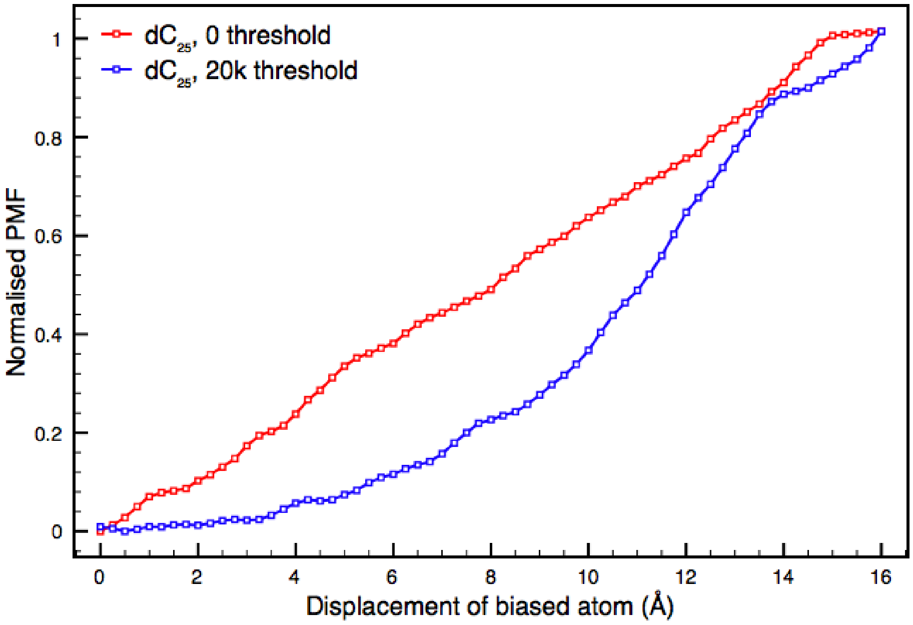

Figure 8: Free energy plots comparing A_{25} and dC_{25} ABF translocation simulations with ζ values of 0 and 20000. The free energy has been normalised so that the x-axis end points are the same for each profile to illustrate the difference in the profile shapes. The distinctive curved profile shape that is present when ζ is 20000 is absent when ζ is 0.

has on the estimated free energy profile by determining the free energy difference across the reaction coordinate at several values of ζ, and extrapolating what the free energy difference might be as $\zeta \to \infty$ (i.e. an unbiased simulation). This may be more difficult with higher ζ values due to the fluctuations in translocation progression and the impact this has on the free energy profile.

10 shows free energy profiles from single sample ABF simulations of dC_1 translocation at ζ values of 5000 and 80000, plotted with the force measurements per bin taken to calculate the biasing force for that bin. Unlike the polynucleotide case, the single nucleotide free energy profiles show less dependence on the force measurements per bin. The plots display well defined shapes at both values of ζ, regardless of the number of force measurements per bin in the corresponding region. Intuitively we can expect that single nucleotide molecules have faster relaxing degrees of freedom than polynucleotides, accounting for this trend. The plot shows a decrease in the cumulative free energy value as ζ increases, though unlike the polynucleotide case, a significant change in shape is not observed at ζ=80000. A plateau in both of the profiles occurs after roughly 10 Å as was observed in the cv-SMD profiles of the same system. This is in line with the expected influence of the protein constriction on the translocating molecule.

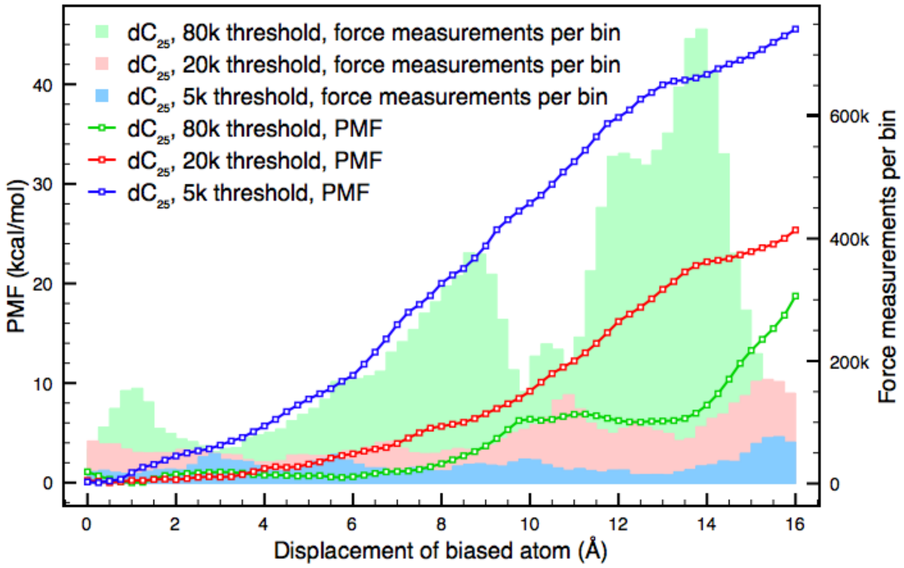

Figure 9: Free energy profiles from single sample ABF simulations of dC_{25} translocation at ζ values of 5000, 20000, and 80000. The histograms represent the number of instantaneous force measurements per bin. The variation in sampling is roughly proportional to the ζ value.

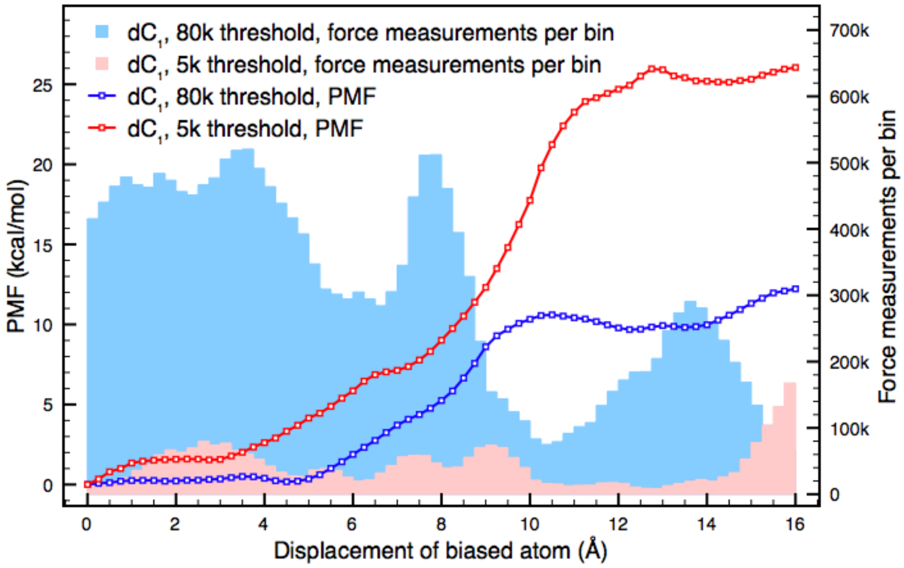

Figure 10: Free energy profiles from single sample ABF simulations of dC_1 translocation at ζ values of 5000 and 80000. The histograms represent the number of instantaneous force measurements per bin. The shape is not attributed to the influence of the αHL constriction nor the variation in force measurements per bin.

References

[1] L. Kale, R. Skeel, M. Bhandarkar, R. Brunner, A. Gursoy, N. Krawetz, J. Phillips, A. Shinozaki, K. Varadarajan, and K. Schulten. NAMD2: Greater scalability for parallel molecular dynamics. *J. Comp. Phys.*, 151(1):283–312, 1999.

[2] C. Jarzynski. Nonequilibrium Equality for Free Energy Differences. *Phys. Rev. Lett.*, 78(14):2690–2693, 1997.

[3] E. Darve and A. Pohorille. Calculating free energies using average force. *J. Chem. Phys.*, 115:9169, 2001.

[4] E. Darve, M.A. Wilson, and A. Pohorille. Calculating free energies using a scaled-force molecular dynamics algorithm. *Mol. Sim.*, 28(1-2):113–144, 2002.

[5] J. Hénin and C. Chipot. Overcoming free energy barriers using unconstrained molecular dynamics simulations. *J. Chem. Phys.*, 121:2904, 2004.

[6] C. Chipot and J. Hénin. Exploring the free-energy landscape of a short peptide using an average force. *J. Chem. Phys.*, 123:244906, 2005.

[7] J. Hénin, G. Fiorin, C. Chipot, and M.L Klein. Exploring Multidimensional Free Energy Landscapes Using Time-Dependent Biases on Collective Variables. *J. Chem. Theor. Comp.*, 6(1):164–170, 2010.

[8] A. Aksimentiev and K. Schulten. Imaging α-Hemolysin with Molecular Dynamics: Ionic Conductance, Osmotic Permeability, and the Electrostatic Potential Map. *Biophys. J.*, 88(6):3745–3761, 2005.
